# Single-Photon Emission Computer Tomography Imaging of Prostate-Specific Membrane Antigen (PSMA) Expression in Prostate Cancer Patients Using a Novel Peptide-Based Probe [^99m^Tc]Tc-BQ0413 with Picomolar Affinity to PSMA: A Phase I/II Clinical Study

**DOI:** 10.1021/acsptsci.4c00637

**Published:** 2025-02-21

**Authors:** Anna Medvedeva, Vladimir Chernov, Maria Larkina, Anastasiya Rybina, Roman Zelchan, Olga Bragina, Ruslan Varvashenya, Olga Zebzeeva, Ekaterina Bezverkhniaia, Vladimir Tolmachev, Anna Orlova

**Affiliations:** † Department of Nuclear Therapy and Diagnostic, Cancer Research Institute, Tomsk National Research Medical Center, Russian Academy of Sciences, 634009 Tomsk, Russia; ‡ Research Centrum for Oncotheranostics, Research School of Chemistry and Applied Biomedical Sciences, Tomsk Polytechnic University, 634009 Tomsk, Russia; § Department of Pharmaceutical Analysis, Siberian State Medical University, 634050 Tomsk, Russia; ∥ Department of Medicinal Chemistry, Uppsala University, 751 23 Uppsala, Sweden; ⊥ Department of Immunology, Genetics and Pathology, Uppsala University, 752 37 Uppsala, Sweden; # Science for Life Laboratory, Uppsala University, 751 23 Uppsala, Sweden

**Keywords:** prostate cancer, PSMA, SPECT, phase I/II

## Abstract

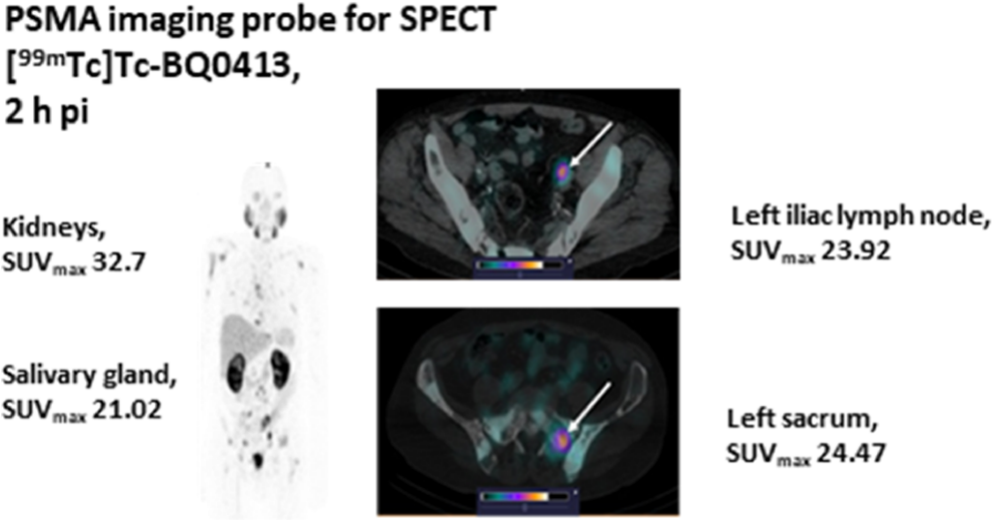

Radionuclide imaging of prostate-specific membrane antigen (PSMA) expression can be used for staging prostate cancer. The pseudo-peptide [^99m^Tc]Tc-BQ0413 demonstrated high affinity and specificity to PSMA in preclinical evaluation. The purpose of this study was to clinically evaluate the safety and tolerability of a single administration of [^99m^Tc]Tc-BQ0413 as well as study its biodistribution using SPECT to estimate dosimetry. [^99m^Tc]Tc-BQ0413 was studied in a single-center diagnostic Phase I open-label exploratory study. Whole-body planar scintigraphy and SPECT/CT imaging were performed 2, 4, and 6 h after administration of 50, 100, or 150 μg (680 ± 140 MBq) of [^99m^Tc]Tc-BQ0413 in five PCa patients per injected mass (NCT05839990). All injections of [^99m^Tc]Tc-BQ0413 were well tolerated. The elimination of [^99m^Tc]Tc-BQ0413 was predominantly renal. The stable physiological uptake of [^99m^Tc]Tc-BQ0413 was observed in the lacrimal and salivary glands, liver, spleen, and kidneys for all tested peptide-injected masses. The average effective doses were 0.007 ± 0.001, 0.0049 ± 0.0003, and 0.0062 ± 0.0008 mSv/MBq for 50, 100, and 150 μg/injection, respectively. The radionuclide-associated dose burden per patient was 4–7 mSv/study for the given activity. Uptake of [^99m^Tc]Tc-BQ0413 in primary tumors was identified in all patients and increased with the peptide-injected mass. Uptake in lymph nodes and bone metastases was the highest at 100 μg/injected mass. The highest tumor lesion/background ratios were observed 6 h after the administration of 100 μg of [^99m^Tc]Tc-BQ0413. The results of the Phase I study showed that injections of [^99m^Tc]Tc-BQ0413 were well tolerated, safe, and associated with low absorbed doses. Imaging using [^99m^Tc]Tc-BQ0413 enabled the visualization of primary prostate cancer lesions as well as metastases in lymph nodes and bones.

Prostate cancer (PCa) is the second most diagnosed cancer form worldwide, and about one in eight men will be diagnosed with PCa during their lifetime.^1^ About 1.5 million men were diagnosed with PCa in 2020 and despite the overall incidence rate increasing by 3% each year, the mortality from PCa dropped by half between 1993 and 2013 and continues to decline slowly.^2^ These achievements should be attributed to improvements in therapy and patient management.

The diagnosis of PCa is a multistep process and includes the analysis of several biomarkers in blood and urine probes as well as digital rectal examination and imaging investigations, all aiming to “minimize unnecessary biopsies” for low-risk patients and “maximize cancer detection” for high-risk patients. In the case of suspected clinically relevant PCa, pathohistological analysis of biopsy material can confirm the presence of PCa.^3^ Nuclide-based imaging, positron emission tomography (PET), and single-photon emission computer tomography (SPECT) are recommended by the American Society of Clinical Oncology (ASCO) for patients with newly diagnosed, high-risk PCa, patients with suspected or confirmed metastasized cancer, and patients with recurrent cancer and therapy resistance.^4^ Different imaging probes approved by the FDA can be used for the detection of cancer spread, including [^99m^Tc]Tc-medronate, [^18^F]F-fluoride, [^11^C]C-choline, and [^18^F]F-fluciclovine. These radiotracers provide higher accuracy in the detection of PCa lesions than [^18^F]F-FDG; however, FDG can be used for the detection of higher-grade, castration-resistant, and neuroendocrine PCa.^3^

Within the last 15 years, the development of pseudo-peptide Glu-urea-Lys-based (EuK) prostate-specific membrane antigen (PSMA) ligands has dramatically changed the diagnosis and staging of PCa.^5^ PSMA is a metallopeptidase that is a suitable and reliable tissue marker due to its high expression on PCa cell membrane and documented overexpression in over 90% of all PCa.^6^ Intensive work on the development of PSMA probes suitable for clinical application resulted in the recent approval of three probes for PET diagnosis: [^68^Ga]Ga-PSMA-11, [^18^F]F-DCFPyL, and Flotufolastat F-18, followed by the radioligand for targeted radiotherapy, Pluvicto (lutetium-177 vipivotide tetraxetan, also known as [^177^Lu]Lu-PSMA-617), for the treatment.^3^ PSMA imaging using PET is a reliable diagnostic tool that provides a detection rate of 95% for patients with PSA levels over 2 ng/mL and is recommended not only for the detection of metastasis, disease progression, and biochemical recurrence but also for initial staging and for planning of targeted radiotherapy using [^177^Lu]Lu-PSMA-617.

The approved probes were developed for PET, which provides high sensitivity. However, SPECT scanners are more widely accessible than PET scanners and have lower investigation costs. Recent statistics from the IAEA show that there are 5-fold more SPECT cameras per 1 million than PET cameras.^7^ More imaging procedures are required than current PET availability could provide, taking into account the incidence rate for PCa and the aging population. Furthermore, the development of a new generation of SPECT cameras has improved their sensitivity.

The development of PSMA probes suitable for SPECT, labeled with Tc-99m, I-123, or In-111, was not as intensive compared to probes suitable for PET. No PSMA-targeting SPECT imaging probes are approved for clinical use yet.^8^ There are PSMA-targeting probes containing DOTAGA chelator, PSMA-617, and PSMA I&T that can be labeled with indium-111; however, the low availability and long half-life of this nuclide diminish its attractiveness for clinical use.^9^ Two PSMA-targeting probes labeled with iodine-123 were tested clinically: [^123^I]-MIP-1072 and [^123^I]-MIP-1095.^10^ The radioiodination of the probes offers the application of theranostic concept: iodine-123 can be used for SPECT and iodine-124 for PET diagnostic investigations, while iodine-131 for systemic therapy. However, radioiodination is a complex process including multiple synthetic and purification steps.^11^

The most attractive SPECT isotope is technetium-99m. It is cheap and widely available due to the use of a ^99^Mo generator. Its labeling chemistry for medical applications has been developed,^12^ and Tc-99m-based probes account for 80% of all imaging procedures in nuclear medicine representing 30–40 million examinations annually worldwide.^13^ Several PSMA imaging probes labeled with Tc-99m have been developed and tested clinically within the past decade.^14−16^ PSMA imaging using SPECT demonstrated good accuracy in the detection of PCa lesions at both staging and recurrence.^17^ The comparison of visualization of PSMA-positive PCa lesions using PSMA SPECT/CT demonstrated similar detection rate for prostate, seminal vesicles, and visceral lesions, and 85−90% low rate for detection of metastases in locoregional and non-locoregional lymph nodes, and bone metastases to PSMA PET/CT.^18,19^ Imaging of PSMA expression using later SPECT/CT scans increases the detection rate for small metastases, as shown for [^99m^Tc]Tc-PSMA-I&S SPECT/CT.^20^

Different labeling methods have been applied in the preparation of PSMA imaging probes. Initially, the PSMA ligands MIP-1404 and MIP-1405 were labeled with the tricarbonyl complex of Tc-99m.^21,22^ These probes rapidly localized in bone and lymph node lesions with high contrast;^22^ however, labeling of peptides with the Tc(CO)_3_ core is a two-step procedure that requires product purification, which could hinder its clinical implementation.

The search for PSMA imaging probes suitable for one-pot labeling kits has led to the development of HYNIC-containing probes.^14^ Clinical data demonstrated high sensitivity for [^99m^Tc]Tc-HYNIC-PSMA in the detection of PCa lesions with faster blood clearance and lower effective dose for investigation than for MIP-1404 and MIP-1405.^23,24^ This labeling strategy allows a one-step synthesis that is suitable at room temperature; however, the final products are often affected by significant isomerism, and its stability depends on the coligand and labeling conditions.^16^

The direct incorporation of a Tc=O core into the precursor is a simple method suitable for kit preparation. This method provides high radiochemical yields and has been used for the preparation of many radiopharmaceuticals,^13^ including [^99m^Tc]Tc-PSMA-I&S. The probe is produced with consistently high radiochemical yield and purity, is very stable in blood circulation, and demonstrates efficient uptake in PCa lesions; however, it has high binding to plasma protein, resulting in a slow whole-body clearance.^9^ The mentioned limitations of the existing SPECT probes for the visualization of PSMA expression thus justify further development. Indeed, an intensive search for probes targeting somatostatin receptors, despite the clinical use of ^111^In-pentetreotide (OctreoScan, FDA-approved 1994), led to the development of new EMU and FDA-approved somatostatin-targeting theranostics based on DOTATOC and DOTATATE.^25,26^

Recently, our group developed and preclinically characterized a peptide-based probe for SPECT imaging of PSMA expression, [^99m^Tc]Tc-BQ0413.^27^ The imaging probe consists of a PSMA-binding moiety based on a EuK ligand, an affinity enhancement 2-napththyl-l-alanine, and l-tyrosine linker,^28^ and a negatively charged mercapto acetyl-triglutamate chelator (maE3) for labeling with technetium-99m (Figure 1).

**Figure 1:**
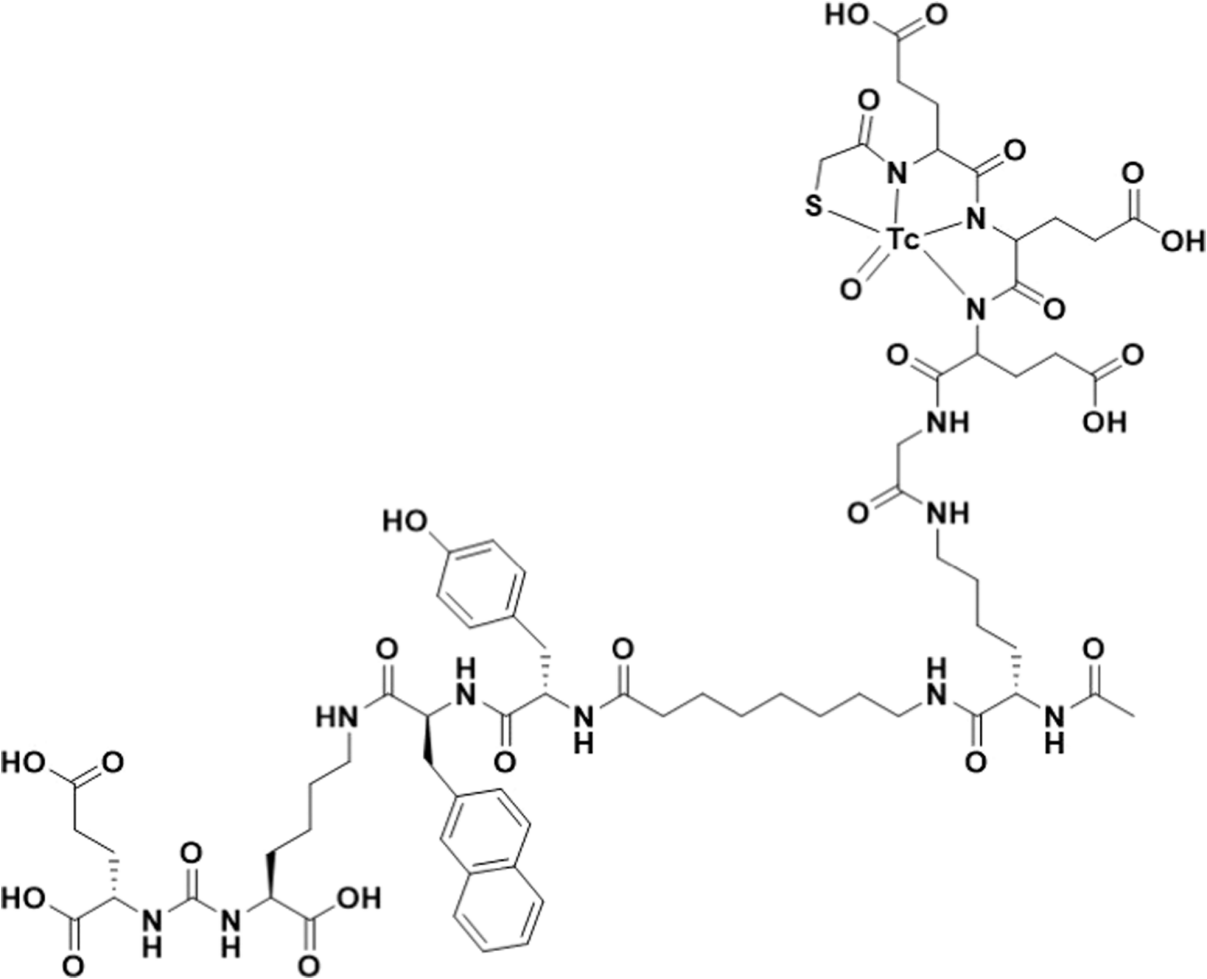
Structure of [^99m^Tc]Tc-BQ0413.

When labeled with technetium-99m, the probe bound specifically to PSMA with high affinity (33 ± 15 pM). [^99m^Tc]Tc-BQ0413 demonstrated PSMA visualization in mice bearing PCa xenografts with a contrast similar to that provided by the PET probe [^68^Ga]Ga-PSMA-11.

In this paper, we report the results of a single-center diagnostic phase I open-label exploratory study of [^99m^Tc]Tc-BQ0413 in PCa patients.

## Results and Discussion

The aim of this study was to conduct a phase I clinical evaluation of [^99m^Tc]Tc-BQ0413 in patients with PCa. Particularly, the aims were to test the tolerability and safety of an intravenous bolus injection of [^99m^Tc]Tc-BQ0413, to study the biological distribution of the labeled peptide after administration of 50, 100, or 150 μg, and to estimate dosimetry.

### Patients

Fifteen patients with prostatic acinar adenocarcinoma were recruited for this Phase I study (Table 1). Examination of biopsy material confirmed PCa in all patients with a Gleason score (GS) of 6–10; the median PSA level was 6.67 [0.006–5000]. At the time of inclusion in the study, 14 patients had a primary tumor, five patients did not have clinical evidence of metastatic disease, one patient had metastases only in regional lymph nodes, one had only a single bone metastasis, two patients had multiple metastases in lymph nodes, and seven had multiple distant bone and lymph node metastases.

**Table 1:** Patient Characteristics

peptide-injected mass, μg	patient no	age, *y*	histotype PSA^a^	clinical stage	phase of the disease
50	P1	70	PAA G5	T4N1M0	HS
			GS 10 (5 + 5)		
			PSA = 0.1		
	P2	55	PAA G5	T4N1M1	HS
			GS 9 (4 + 5)		
			PSA = 0.01		
	P3	64	PAA G4	T4N1M0	HS
			GS 8 (4 + 4)		
			PSA = 9.3		
	P4	70	PAA G4	T1bN0M1	CR
			GS 8 (4 + 4)		
			PSA = 1.52		
	P5	76	PAA G5	T3N1M1	CR
			GS 9 (4 + 5)		
			PSA = 5000		
100	P6	65	PAA G2	T4N1M1	CR
			GS 7 (3 + 4)		
			PSA = 188		
	P7	64	PAA G5	T4N1M1	CR
			GS 10 (5 + 5)		
			PSA = 6.67		
	P8	55	PAA G2	T4N1M1	CR
			GS 7 (3 + 4)		
			PSA = 22		
	P9^b^	61	PAA G2	T2N0M0	CR
			GS 7 (3 + 4)		
			PSA = 0.3		
	P10	62	PAA G4	T2N0M0	HS
			GS 8 (4 + 4)		
			PSA = 14		
150	P11	65	PAA G4	T2N0M0	HS
			GS 8 (4 + 4)		
			PSA = 5.59		
	P12	65	PAA G1	T2N1M1	HS
			GS 6 (3 + 3)		
			PSA = 0.006		
	P13	70	PAA G4	T4N1M1	HS
			GS 8 (4 + 4)		
			PSA = 0.008		
	P14	74	PAA G4	T2N0M0	HS
			GS 8 (5 + 3)		
			PSA = 7.5		
	P15	74	PAA G1	T2N0M0	HS
			GS L7 (3 + 4)/R6(3 + 3)		
			PSA = 150		

a Histological diagnosis: PAA, prostatic acinar adenocarcinoma; Gx, ISUP grade group; GS, Gleason score; PSA, prostate-specific antigen concentration in blood, ng/mL; HS, hormone-sensitive; CR, castration-resistant.

b The prostate gland was removed.

#### Overall Distribution of [^99m^Tc]Tc-BQ0413, Safety, and Tolerability for Single Intravenous Bolus Injection of [^99m^Tc]Tc-BQ0413

The patients tolerated the injections well. No adverse events, abnormal clinical laboratory findings, or vital sign changes were observed following a single intravenous bolus administration of [^99m^Tc]Tc-BQ0413 at injected masses of 50, 100, and 150 μg.

**Figure 2:**
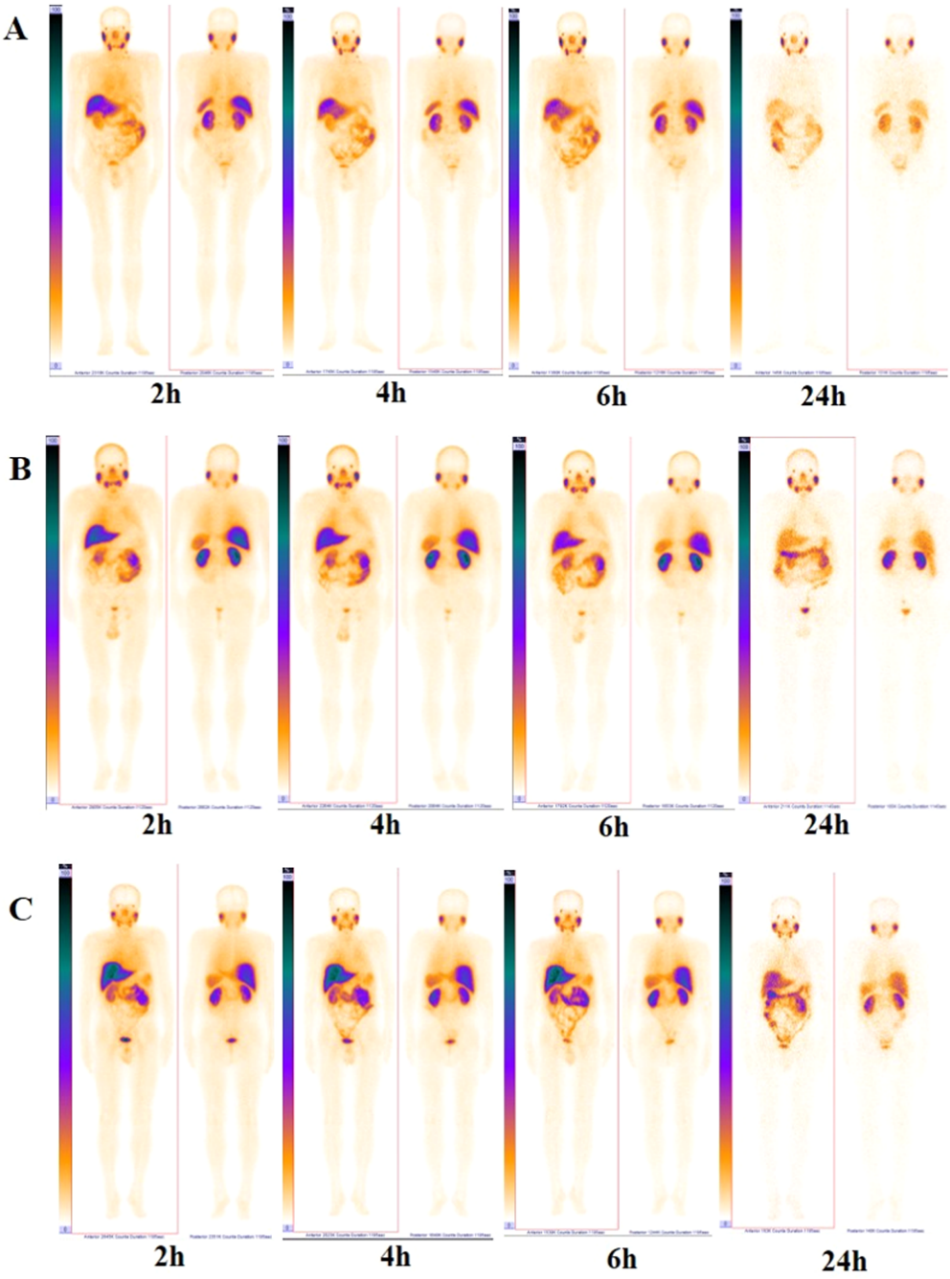
Representative anterior and posterior images of patients with PCa 2, 4, and 6 h after administration of 50 (A), 100 (B), and 150 μg (C) of [^99m^Tc]Tc-BQ0413.

The distribution of [^99m^Tc]Tc-BQ0413 throughout the body of patients at different dosages (50, 100, and 150 μg) did not differ visually on planar images (Figure 2). Two hours after the administration of the radiopharmaceutical, its accumulation was observed in the lacrimal and salivary glands, liver, kidneys, spleen, urinary bladder, and fragmentarily in the intestine. Decay-corrected activity accumulation in the salivary glands, spleen, excretory organs, liver, and kidneys was stable over time, with a tendency to increase for salivary glands and decrease in the liver (Figure S1). Accumulation of activity in anatomically relevant organs, bones, and muscles was much lower than that in excretory organs and organs with endogenous expression of PSMA.

**Figure 3:**
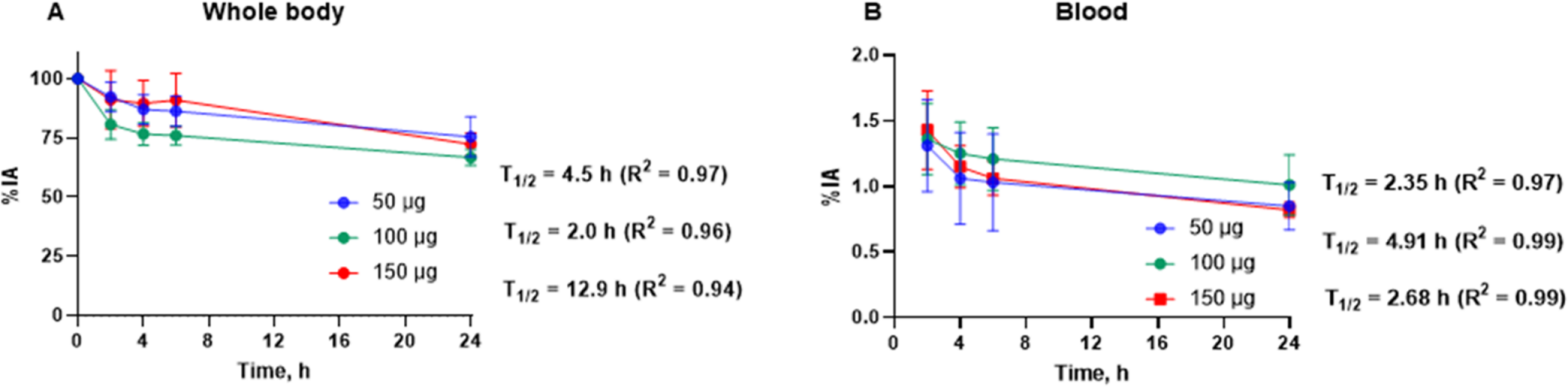
Kinetics of whole-body elimination (A) and blood clearance (B) of [^99m^Tc]Tc-BQ0413. Data were decay-corrected.

The elimination of [^99m^Tc]Tc-BQ0413 was relatively slow, similar to [^99m^Tc]Tc-MIP-1404,^22^ and about 75% of injected activity (%IA, decay-corrected) was not excreted 24 h after administration (Figure 3A). The half-life of the elimination phase was the longest for the 150 μg peptide-injected mass (12.9 h), while for lower injected masses, it was 3–6-fold shorter. Clearance from blood circulation was very similar for all peptide-injected masses (Figure 3B).

#### Dosimetry Assessment

The absorbed doses for healthy organs were calculated using whole-body images at 4 time points, and the results are presented in Table 2. The absorbed doses were the lowest for injection of 100 μg of peptide in the majority of healthy organs, with a few exceptions. The absorbed dose in the kidneys was the highest at 100 μg; in the spleen, the absorbed dose tended to increase with increasing peptide mass, while in the lungs and thyroid, the absorbed dose tended to decrease. The effective dose was the lowest for 100 μg of the injected peptide. The dose burden values were 4.4 ± 1.1, 3.2 ± 0.5, and 3.9 ± 0.5 mSv per single investigation with 50, 100, and 150 μg of [^99m^Tc]Tc-BQ0413.

**Table 2:** Absorbed Doses (mGy/MBq) after iv Injection of [^99m^Tc]Tc-BQ0413

target organ	50 μg	100 μg	150 μg
adrenals	0.0166 ± 0.0102	0.0089 ± 0.0022	0.0113 ± 0.0027
brain	0.0017 ± 0.0006	0.0013 ± 0.0001	0.0016 ± 0.0002
breasts	0.0018 ± 0.0006	0.0014 ± 0.0002	0.0016 ± 0.0001
gallbladder wall	0.0095 ± 0.0033	0.0063 ± 0.0004	0.0083 ± 0.0004
lower large-intestine wall	0.0075 ± 0.0025	0.0046 ± 0.0017	0.0066 ± 0.0013
small intestine	0.0065 ± 0.0009	0.0051 ± 0.0005*	0.0067 ± 0.0008
stomach wall	0.0054 ± 0.0003**^a^	0.0041 ± 0.0003***	0.0058 ± 0.0008
upper large-intestine wall	0.0067 ± 0.0012*	0.0047 ± 0.0007*	0.0068 ± 0.0007
heart wall	0.0075 ± 0.0009	0.0071 ± 0.0009	0.0075 ± 0.0007
kidneys	0.0267 ± 0.0151	0.0321 ± 0.0094	0.0247 ± 0.0101
liver	0.0147 ± 0.0020*	0.0108 ± 0.0007**	0.0160 ± 0.0029
lungs	0.0078 ± 0.0044	0.0063 ± 0.0019	0.0056 ± 0.0014
muscle	0.0025 ± 0.0004	0.0020 ± 0.0001*	0.0026 ± 0.0002
pancreas	0.0085 ± 0.0010*	0.0062 ± 0.0008*	0.0091 ± 0.0019
red marrow	0.0036 ± 0.0006	0.0029 ± 0.0002	0.0036 ± 0.0003
osteogenic cells	0.0085 ± 0.0017	0.0068 ± 0.0006	0.0088 ± 0.0010
skin	0.0020 ± 0.0004	0.0016 ± 0.0001	0.0020 ± 0.0002
spleen	0.0163 ± 0.0061	0.0168 ± 0.0060	0.0209 ± 0.0067
testes	0.0048 ± 0.0021	0.0028 ± 0.0005	0.0053 ± 0.0019
thymus	0.0104 ± 0.0045	0.0053 ± 0.0015	0.0080 ± 0.0030
thyroid	0.0135 ± 0.0090	0.0087 ± 0.0039	0.0094 ± 0.0024
urinary bladder wall	0.0061 ± 0.0012	0.0046 ± 0.0009	0.0073 ± 0.0036
prostate	0.0048 ± 0.0008	0.0034 ± 0.0006	0.0047 ± 0.0011
parotid salivary gland^b^	0.03 ± 0.01	0.025 ± 0.01	0.029 ± 0.009
total	0.0039 ± 0.0006	0.0031 ± 0.0002*	0.0040 ± 0.0003
effective dose equivalent (mSv/MBq)	0.0088 ± 0.0015	0.0072 ± 0.0008	0.0083 ± 0.0008
effective dose (mSv/MBq)	0.0067 ± 0.0011*	0.0049 ± 0.0004	0.0062 ± 0.0008

a Results of ordinary one-way ANOVA with Bonferroni correction are presented as *p < 0.05, **p < 0.01, and ***p < 0.001. A statistically significant difference between groups injected with 50 and 150 μg of the peptide is marked in column 50 μg, and the difference between groups injected with 100 and 150 μg of the peptide is marked in column 100 μg.

b Doses for the parotid salivary gland were calculated based on the SUV using a sphere model.

#### Analyses of Images

**Figure 4:**
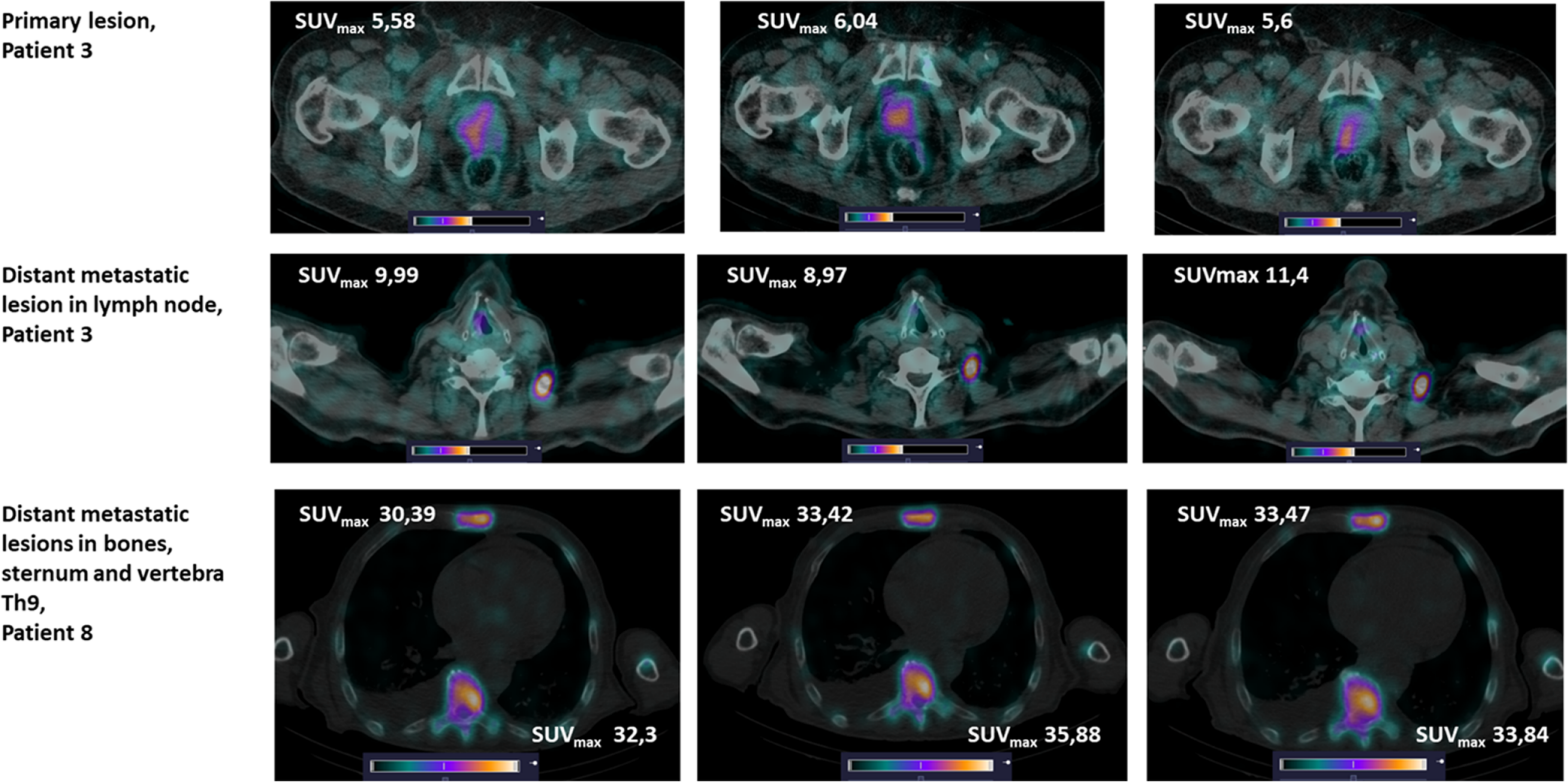
Combined SPECT/CT images of primary lesions and distant metastatic lesions in LN, and bones at 2, 4, and 6 h (left to right) after administration of [^99m^Tc]Tc-BQ0413. The upper setting of the scale window (45% of the maximum scale value for patient 3 and 100% of the maximum scale value for patient 8) was adjusted to clearly visualize the lesion.

The imaging findings of SPECT/CT are summarized in Table 3 and Figure 4. Fourteen of the 14 primary tumors were visualized already 2 h after the administration of [^99m^Tc]Tc-BQ0413. The maximum standardized uptake values (SUV_max_) for primary lesions did not change with time, but the mean SUV (SUV_mean_) demonstrated a tendency to increase with the increase of injected peptide mass for all three time points. In nine patients with initially diagnosed metastases in lymph nodes (LN), lesions were visualized in seven patients. Known LN metastases were not visualized in two patients; both were administered 150 μg of the peptide. The SUV_max_ values for metastases in LN demonstrated a tendency to increase with time after administration of the radiopeptide. The SUV_mean_ values for lesions in LN for the group administered 100 μg of peptide were significantly higher than those for the group administered 50 μg. Distant metastases in bones (BM) were visualized in seven out of eight patients diagnosed with M1-x. The SUV_max_ values were stable over time. The highest SUV_mean_ was found in the 100 μg group, and the lowest was found in the 150 μg group. No lesion-to-lesion analysis was performed.

**Table 3:** Imaging Findings in PCa Patients Administered [99 mTc]Tc-BQ0413

			SUV_max_			average SUV_mean_		
peptide-injected mass, μg	patient no	lesion^a^	2 h	4 h	6 h	2 h	4 h	6 h
50	P1	PT	3.71	3.55	3.64	PT 3.4 ± 1.4 [1.15–4.82]	PT 3.2 ± 1.5 [1.07–5.36]	PT 3.4 ± 1.6 [0.83–4.92]
		LNM	4.04	4.29	4.89	LNM 6.1 ± 4.1 [1.72–10.19]	LNM 6.2 ± 4.1 [1.87–11.1]	LNM 6.4 ± 4.0 [1.86–9.99]
		BM	ND^b^			BM 14 ± 16 [2.39–22.12]	BM 12 ± 15 [1.83–22.36]	BM 12 ± 14 [2.02–22.03]
	P2	PT	4.03	3.98	5.23			
		LNM	1.96	2.1	2.08			
		BM	ND					
	P3	PT	5.58	6.07	5.6			
		LNM	9.99	8.97	11.49			
		BM	ND					
	P4	PT	4.32	3.65	3.86			
		LNM	ND					
		BM	2.7	2.03	2.26			
	P5	PT	1.35	1.21	0.96			
		LNM	11.62	12.58	10.87			
		BM	25.11	25.28	24.95			
100	P6	PT	7.91	8.27	6.75	PT 11.5 ± 15.6 [1.42–34.71]	PT 11.3 ± 14.9 [1.43–33.3]	PT 12.2 ± 17.2 [1.69–37.6]
		LNM	15.33	17.55	15.22	LNM 29.6 ± 28.9 [12.34–62.99]	LNM 31.5 ± 30.9 [11.87–67.16]	LNM 36.3 ± 37.2 [13.38–79.28]
		BM	28.55	29.84	28.72	BM 20.0 ± 12.3 [6.41–30.29]	BM 21.6 ± 12.3 [7.67–30.91]	BM 22.5 ± 13.4 [7.88–34.26]
	P7	PT	39.61	37.87	42.85			
		LNM	14.01	13.47	18.67			
		BM	7.25	8.74	8.96			
	P8	PT	1.58	1.58	1.94			
		LNM	72.18	76.27	89.22			
		BM	35.04	36.14	39.17			
	P9	PT	NA					
		LNM	ND					
		BM	ND					
	P10	PT	3.4	3.66	3.83			
		LNM	ND					
		BM	ND					
150	P11	PT	6.11	5.48	6.12	PT 5.1 ± 1.2 [3.86–7.01]	PT 5.1 ± 2.0 [3.4–8.16]	PT 5.5 ± 3.0 [2.07–9.84]
		LNM	ND			LNM N/A	LNM N/A	LNM N/A
		BM	ND			BM 0.88 ± 0.35 [0.63–1.12]	BM 1.7 ± 1.0 [0.97–2.39]	BM 1.2 ± 1.1 [0.41–1.96]
	P12	PT	4.76	3.99	4.13			
		LNM	ND					
		BM	1.28	2.7	2.19			
	P13	PT	4.4	3.4	2.41			
		LNM	ND					
		BM	0.7	1.07	0.47			
	P14	PT	7.96	9.3	11.19			
		LNM	ND					
		BM	ND					
	P15	PT	6.03	6.76	7.29			
		LNM	ND					
		BM	ND					

a PT, prostate tumor; LNM, lymph node metastases; and BM, bone metastases.

b ND, not detected; NA, not applicable.

**Figure 5:**
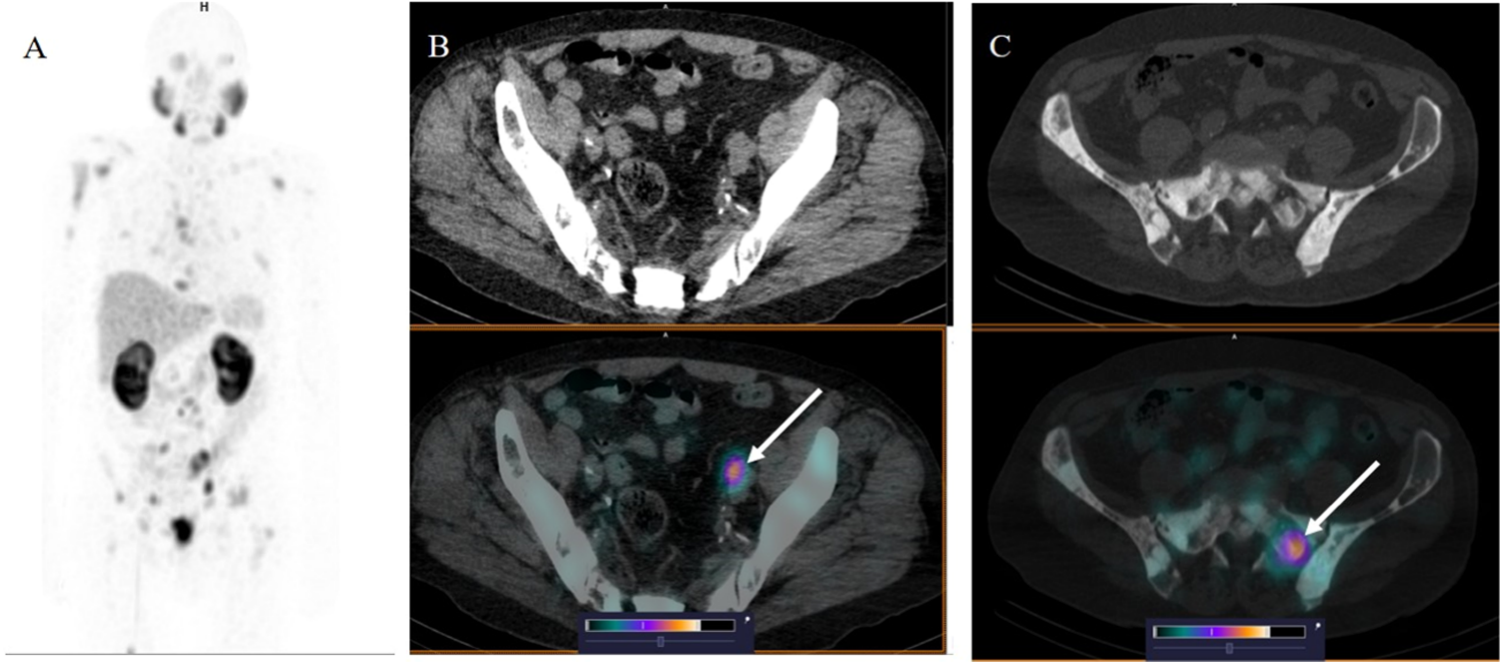
SPECT image 2 h after injection of [^99m^Tc]Tc-BQ0413 at an injected mass of 100 μg in a patient with castration-resistant prostate cancer with multiple metastases in the bones and lymph nodes (patient 7). (A) SPECT 3-dimensional maximum-intensity projection, front view (30% of the maximum scale value); (B) radiopeptide accumulation in the left iliac lymph node, SUV_max_ 23.92; and (C) radiopeptide accumulation in the left sacrum SUV_max_ 24.47 (73% of the maximum scale value).

High-contrast images were obtained 2 h after administration of [^99m^Tc]Tc-BQ0413 at all peptide-injected masses (Figure 5). The SUV_mean_ values for muscle and bones, the anatomically relevant normal tissue, were below 1 for all tested peptide-injected masses and time points (Table 4). The highest SUV_mean_ values among normal organs were found in the kidneys, followed by the salivary glands, liver, and spleen. The SUV_mean_ values for salivary glands increased with time, while for other organs with high activity accumulation, SUV_mean_ values were stable over time. The group injected with 100 μg of the peptide demonstrated a higher activity uptake in the kidneys and a lower uptake in the liver.

**Table 4:** Mean Standardized Uptake Values (SUV_mean_) for Different Normal Organs over Time in PCa Patients Administered [^99m^Tc]Tc-BQ0413

		time after administration		
administered peptide-injected mass (μg)	organ	2 h	4 h	6 h
50	bone	0.7 ± 0.1	0.6 ± 0.2	0.4 ± 0.1
	muscle	0.9 ± 0.3	0.8 ± 0.4	0.6 ± 0.3
	lung	1.4 ± 0.2	1.3 ± 0.4	1.3 ± 0.3
	kidney	21 ± 6	20 ± 6	21 ± 7
	salivary gland	16 ± 8	17 ± 7	20 ± 10
	liver	13 ± 2	12 ± 3	11 ± 3
	spleen	11 ± 5	10 ± 5	10 ± 4
100	bone	0.5 ± 0.1	0.6 ± 0.2	0.5 ± 0.2
	muscle	0.8 ± 0.3	0.7 ± 0.1	0.6 ± 0.2
	lung	1.3 ± 0.2	1.3 ± 0.3	1.2 ± 0.3
	kidney	27 ± 2	29 ± 4	29 ± 6
	salivary gland	17 ± 7	19 ± 7	20 ± 9
	liver	11 ± 2	10 ± 2	10 ± 2
	spleen	10 ± 4	10 ± 3	10 ± 3
150	bone	0.7 ± 0.2	0.9 ± 0.3	0.6 ± 0.2
	muscle	0.8 ± 0.4	0.8 ± 0.2	0.6 ± 0.2
	lung	1.1 ± 0.2	0.9 ± 0.4	1.0 ± 0.1
	kidney	22 ± 3	22 ± 4	22 ± 4
	salivary gland	16 ± 7	18 ± 6	19 ± 6
	liver	13 ± 3	13 ± 3	12 ± 2
	spleen	10 ± 3	11 ± 3	11 ± 3

The ratios of SUV_mean_ values for cancer lesions and relevant normal tissue (primary tumor/muscle, LNM/muscle, LNM/lungs, and BM/bones) were calculated for each patient (Table 5). All values increased with time. For primary tumors, the ratios increased with an increase in the administered peptide-injected mass. The ratios for the group administered 100 μg were higher than those for the group administered 50 μg. In these two groups, the ratios increased with disease progression: ratios for primary tumors were lower than those for LNM, and ratios for LNM were lower than those for BM. For the 150 μg administered injected mass, no known LNM was detected on SPECT images, and the ratios for BM were low.

**Table 5:** Ratios of SUV_mean_ Values for Cancer Lesions and Relevant Normal Tissue in PCa Patients Administered [^99m^Tc]Tc-BQ0413

injected mass	50 μg			100 μg			150 μg		
time	2 h	4 h	6 h	2 h	4 h	6 h	2 h	4 h	6 h
PT/M^a^	3.8 ± 1.4 [2.3–5.3]	4.6 ± 2.4 [2.2–8.0]	5.6 ± 1.9 [3.8–8.7]	15.3 ± 20.1 [1.2–45.1]	16.8 ± 22.3 [1.6–49.7]	20.6 ± 27.9 [2.0–62.1]	6.5 ± 2.6 [3.7–9.9]	6.7 ± 2.8 [4.0–10.9]	8.4 ± 4.6 [3.2–13.5]
LNM/M	8.8 ± 8.4 [2.6–20.4]	11.9 ± 12.7 [2.6–30.0]	18.5 ± 23.3 [3.5–53.0]	27.9 ± 20.2 [16.0–51.2]	38.7 ± 31.9 [17.7–75.5]	46.0 ± 41.2 [17.8–93.3]			
LNM/L	4.34 ± 3.13 [1.3–7.1]	5.3 ± 4.2 [1.5–9.1]	5.7 ± 4.6 [1.4–11.1]	20.5 ± 19.1 [8.6–42.6]	23.3 ± 19.4 [10.4–45.7]	30.2 ± 31.7 [9.1–66.6]			
BM/B	21.5 ± 2.56 [3.1–33.5]	23.7 ± 29.7 [2.7–44.7]	42.7 ± 55.0 [3.8–81.6]	42.8 ± 28.3 [10.5–62.9]	40.5 ± 35.9 [8.4–79.3]	122.5 ± 164.2 [15.2–311.5]	1.18 ± 0.01 [1.18–1.19]	2.4 ± 0.2 [2.2–2.5]	1.8 ± 1.5 [0.7–2.8]

a PT/M, prostate tumor/muscle; LNM/M, lymph node metastases/muscle; LNM/L, lymph node metastases/lung; and BM/B, bone metastases/bone.

### Discussion

In recent years, interest in clinical imaging of PCa for staging and restaging has dramatically increased because of the introduction of anti-PSMA EuK-based pseudopeptides. Nowadays, clinicians have three approved probes for the visualization of PSMA expression, all dedicated to PET imaging modality. The need for a PSMA imaging probe suitable for SPECT is obvious due to the broader availability of SPECT cameras all over the world and lower costs for SPECT investigation in comparison to PET. The introduction of SPECT PSMA in clinical practice would allow it to reach more PCa patients.

We present the results of the Phase I clinical study for the new PSMA-targeting peptide-based probe for the visualization of PCa lesions, [^99m^Tc]Tc-BQ0413. The peptide-based probe was administered to patients with prostatic acinar adenocarcinoma after a simple and straightforward labeling procedure. Three different injected masses of peptides were tested in this study. All injections were well tolerated, and no adverse events were observed. An increase of the peptide-injected mass was included in this study to investigate if a mass-dependent decrease in activity accumulation in organs with endogenous PSMA expression, such as kidney and glands in the head and neck area, that was observed in preclinical evaluation of [^99m^Tc]Tc-BQ0413 in a murine model of PCa could be translated in humans.^27^ In the preclinical study, activity uptake in healthy tissues significantly decreased when more peptide was administered, while activity uptake in tumors was not influenced. A dramatic 10-fold decrease was observed, particularly in the kidneys, spleen, and salivary glands. However, we were not able to observe the same effect in this study. We speculate that this could be due to one or a combination of different factors. This Phase I clinical study included a limited number of patients. The peptide dose was increased 3-fold, while in the preclinical study, a 100-fold injected mass increase was performed. We also cannot exclude differences in the affinities of BQ0413 for human and murine PSMA.

The overall biodistribution profile of [^99m^Tc]Tc-BQ0413 was similar to the published data for other anti-PSMA probes labeled with technetium-99m, [^99m^Tc]Tc-MIP-1404, [^99m^Tc]Tc-MIP-1405, [^99m^Tc]Tc-EDDA/HYNIC-iPSMA, and [^99m^Tc]Tc-PSMA I&S.^22,24,29^ The tested agent accumulated in the liver, spleen, kidneys, lacrimal, and salivary glands and had predominantly renal excretion. The exact comparison of published data for other Tc-99m-labeled PSMA probes with our results is not easy due to different experimental conditions (patient cohorts, including PSA values, GS, hormone sensitivity, administered peptide mass, and time points of imaging acquisitions) and data presentation (Table 6). The overall biodistribution profile of [^99m^Tc]Tc-BQ0413, with slow whole-body elimination and elevated and stable over time activity uptake in excretory organs, closely resembled [^99m^Tc]Tc-MIP-1404; however, activity uptake in kidneys and liver was lower for [^99m^Tc]Tc-BQ0413.^22^

**Table 6:** Comparison of Absorbed Doses for Healthy Organs and Tissues for [^99m^Tc]Tc-BQ0413, [^99m^Tc]Tc-MIP-1404, [^99m^Tc]Tc-MIP-1405,^22^ [^99m^Tc]Tc-PSMA I&S,^29^ [^99m^Tc]Tc-EDDA/HYNIC-iPSMA,^24^ and Bone Scintigraphy^37^

probe	[^99m^Tc]Tc-BQ0413			[^99m^Tc]Tc-PSMA I&S	[^99m^Tc]Tc-EDDA/HYNIC-iPSMA	[^99m^Tc]Tc-MIP-1404	[99 mTc]Tc-MIP-1405	bone scan
peptide-injected mass/investigation	50 μg	100 μg	150 μg	25 μg	37–50 μg	100 μg		N/A
activity/investigation, MBq	460–840	560–780	610–755	700	550–740	740		300–740
organ								
adrenals	16.6 × 10^–03^	8.9 × 10^–03^	11.3 × 10^–03^	21.7 × 10^–03^	5.39 × 10^–03^	8.87 × 10^–03^	4.95 × 10^–03^	2.1 × 10^–03^
gallbladder wall	9.5 × 10^–03^	6.3 × 10^–03^	8.3 × 10^–03^	8.22 × 10^–03^	6.22 × 10^–03^	9.70 × 10^–03^	5.54 × 10^–03^	1.4 × 10^–03^
LLI wall	7.5 × 10^–03^	4.6 × 10^–03^	6.6 × 10^–03^	8.90 × 10^–03^	1.84 × 10^–03^	11.6 × 10^–03^	10.8 × 10^–03^	
small intestine	6.5 × 10^–03^	5.1 × 10^–03^	6.7 × 10^–03^	11.9 × 10^–03^	3.27 × 10^–03^	10.4 × 10^–03^	8.30 × 10^–03^	2.3 × 10^–03^
stomach wall	5.4 × 10^–03^	4.1 × 10^–03^	5.8 × 10^–03^	5.00 × 10^–03^	2.89 × 10^–03^	5.74 × 10^–03^	3.72 × 10^–03^	1.2 × 10^–03^
ULI wall	6.7 × 10^–03^	4.7 × 10^–03^	6.8 × 10^–03^	8.49 × 10^–03^	9.45 × 10^–03^	16.1 × 10^–03^	12.5 × 10^–03^	2.3 × 10^–03^
kidneys	26.7 × 10^–03^	32.1 × 10^–03^	24.7 × 10^–03^	73.3 × 10^–03^	38.9 × 10^–03^	73.3 × 10^–03^	36.3 × 10^–03^	7.3 × 10^–03^
liver	14.7 × 10^–03^	10.8 × 10^–03^	16.0 × 10^–03^	12.3 × 10^–03^	14.5 × 10^–03^	16.1 × 10^–03^	6.68 × 10^–03^	1.2 × 10^–03^
red marrow	3.6 × 10^–03^	2.9 × 10^–03^	3.6 × 10^–03^	3.41 × 10^–03^	1.89 × 10^–03^	4.08 × 10^–03^	3.02 × 10^–03^	9.2 × 10^–03^
osteogenic cells	8.5 × 10^–03^	6.8 × 10^–03^	8.8 × 10^–03^	6.45 × 10^–03^	3.52 × 10^–03^	8.36 × 10^–03^	6.44 × 10^–03^	1.0 × 10^–03^
spleen	16.3 × 10^–03^	16.8 × 10^–03^	20.9 × 10^–03^	11.9 × 10^–03^	9.54 × 10^–03^	21.8 × 10^–02^	9.94 × 10^–03^	1.4 × 10^–03^
thyroid	13.5 × 10^–03^	8.7 × 10^–03^	9.4 × 10^–03^	2.62 × 10^–03^	0.655 × 10^–03^	19.5 × 10^–03^	13.3 × 10^–03^	1.3 × 10^–03^
urinary bladder wall	6.1 × 10^–03^	4.6 × 10^–03^	7.3 × 10^–03^	8.89 × 10^–03^	9.98 × 10^–03^	12.7 × 10^–03^	43.4 × 10^–03^	48 × 10^–03^
effective dose (mSv/MBq)	6.7 × 10^–03^	4.9 × 10^–03^	6.2 × 10^–03^	5.2 × 10^–03^	3.73 × 10^–03^	8.78 × 10^–03^	7.87 × 10^–03^	5.7 × 10^–03^
dose burden per single administration	3.08–5.63	2.74–3.82	3.78–4.68	3.11–4.23	2.05–2.76	6.50	5.82	2.9–4.0

High tumor-to-background ratios are required for the detection of small cancer/metastatic lesions. The required ratio is ∼10 for the detection of lesions with about 1 cm diameter, but for the detection of smaller lesions (0.3 cm), the required ratio should be almost 90.^30^ The highest primary tumor-to-background ratios in the present study were observed at 100 μg of the administered peptide at 6 h (46.0 ± 41.2, from 17.72 to 93.27), which was similar to ratios for [^99m^Tc]Tc-PSMA I&S (30.22, from 7 to 110 ^29^) and higher than for [^99m^Tc]Tc-EDDA/HYNIC-iPSMA (from 6.45 to 20.3^24^) and [^99m^Tc]Tc-MIP-1404, [^99m^Tc]Tc-MIP-1405 (around 4^22^). This difference might, to some extent, be explained by the small size and differences in patient cohorts: in the present study, all patients administered 00 μg of the peptide had more advanced PCa than patients administered 50 or 150 μg. The ratios for LNM and BM over the background were also higher in this study than for [^99m^Tc]Tc-EDDA/HYNIC-iPSMA and [^99m^Tc]Tc-PSMA I&S. These comparisons were based on the data reported for Phase I clinical studies when a limited number of unselected PCa patients were included.

It is difficult to make a more detailed comparison between the results of this Phase I study and the performance of the above-mentioned probes that were already tested in larger cohorts of PCa patients. However, [^99m^Tc]Tc-MIP-1404 demonstrated uptake similar to that of [^99m^Tc]Tc-BQ0413 in local recurrences (SUV = 12 ± 7), LNM (20 ± 27), and BM (16 ± 22) in patients with advanced PCa (60 patients with biochemical relapse of PC^31^). The tumor-to-background ratios were 45 ± 36 for local recurrence, 71 ± 69 for LNM, and 37 ± 57 for BM. [^99m^Tc]Tc-EDDA/HYNIC-iPSMA 1 h after administration in patients with metastatic PCa demonstrated higher activity uptake than in the reported Phase I study (23 patients^32^), whose levels were similar to [^99m^Tc]Tc-BQ0413 at 2 h post-administration in this study. The tumor-to-normal tissue ratios were also similar to those obtained in this study. [^99m^Tc]Tc-PSMA I&S in biochemical recurrence or progressive disease performed equally to the Phase I study (72 patients^33^).

The PSMA-targeting probe tested in this study demonstrated the highest affinity among other clinically tested probes for SPECT: for [^99m^Tc]Tc-BQ0413, the value was 33 ± 15 pM;^27^ for [^99m^Tc]Tc-MIP-1404, it was 1.1 ± 0.9 nM; and for [^99m^Tc]Tc-MIP-1405, it was 4.4 ± 0.4 nM.^21^ For [^99m^Tc]Tc-PSMA I&S, the IC_50_ value was 40 ± 1 nM,^9^ and for [^99m^Tc]Tc-EDDA/HYNIC-iPSMA, the affinity value has not yet been reported. A comparison of renal activity accumulation was higher and washout was slower for probes with stronger affinity, [^99m^Tc]Tc-BQ0413, and [^99m^Tc]Tc-MIP-1404,^22^ while moderate affinity led to lower activity accumulation and more efficient washout for [^99m^Tc]Tc-MIP-1405 and [^99m^Tc]Tc-PSMA I&S.^22,29^

A comparison of the imaging performance of [^99m^Tc]Tc-BQ0413 (100 μg, 2 h pi, Tables 3 and 5) with data published for first-in-man or Phase I studies for FDA-approved PET tracers (with all considerations mentioned above) demonstrated that the SPECT probe performed similarly to [^68^Ga]Ga-PSMA-11, [^18^F]F-DCFPyL, and Flotufolastat F-18. Three hours post-administration, SUV_max_ for local recurrences of PCa was 3–39 for [^68^Ga]Ga-PSMA-11, while that for normal prostate gland tissue was 2−4.^34^ The SUV_max_ values 2 h pi of [^18^F]F-DCFPyL were 14 for primary PCa lesion, 26 for LNM, and 46 for BM, while lesion-to-muscle ratios were 12, 38, and 32, respectively.^35^ At the same time point, SUV_max_ for [^18^F]F-rhPSMA-7.3 (Flotufolastat F-18) were 4–19 in primary newly diagnosed PCa, 20 ± 8 for LNM, and 7 ± 5 for BM in castration-resistant metastatic PCa.^36^ The overall activity biodistribution profile was also similar for [^99m^Tc]Tc-BQ0413 and PET tracers.

One of the objectives of this study was to determine the absorbed dose in normal organs and tissues and the effective dose per investigation (Table 2). The absorbed doses for individual organs and effective dose were rather similar to all three tested peptide-injected masses; however, the absorbed doses (except the dose for kidneys), as well as the effective dose, were lower at 100 μg injected mass, which corroborates the data for biodistribution. The organs with the highest absorbed dose were the kidneys for all tested injected masses (0.027 ± 0.015, 0.032 ± 0.010, and 0.025 ± 0.010 mGy/MBq for 50, 100, and 150 μg, respectively) and salivary glands (0.03 ± 0.01, 0.025 ± 0.01, and 0.029 ± 0.009 mGy/MBq for 50, 100, and 150 μg, respectively). Increased absorbed doses were found in the spleen (0.016–0.021 mGy/MBq), adrenal glands (0.009–0.017 mGy/MBq), liver (0.011–0.016 mGy/MBq), and thyroids (0.009–0.014 mGy/MBq). Effective doses were 0.007 ± 0.001, 0.0049 ± 0.0004, and 0.0062 ± 0.0008 mSv/MBq for 50, 100, and 150 μg, respectively. A comparison of absorbed doses and effective doses with available data for PSMA imaging probes for SPECT (Table 6) demonstrates that a single investigation using [^99m^Tc]Tc-BQ0413 will give an effective dose close to or even lower than that for the other probes and will be similar to the dose from a bone scan. Despite renal excretion and elevated activity uptake in the kidneys, [^99m^Tc]Tc-BQ0413 demonstrated the lowest absorbed dose for the kidneys and urinary bladder among the PSMA-targeting probes, which, however, did not translate into higher absorbed doses for the liver and abdominal organs.

In conclusion, in this study, we demonstrated that the administration of a new peptide-based probe [^99m^Tc]Tc-BQ0413 for imaging PSMA expression in PCa was safe and well tolerated. The absorbed doses for the healthy organs and tissues were low, and the effective dose as well as dose burden per single administration corresponded to those for the bone scan.

## Materials and Methods

### Patients

**Figure 6:**
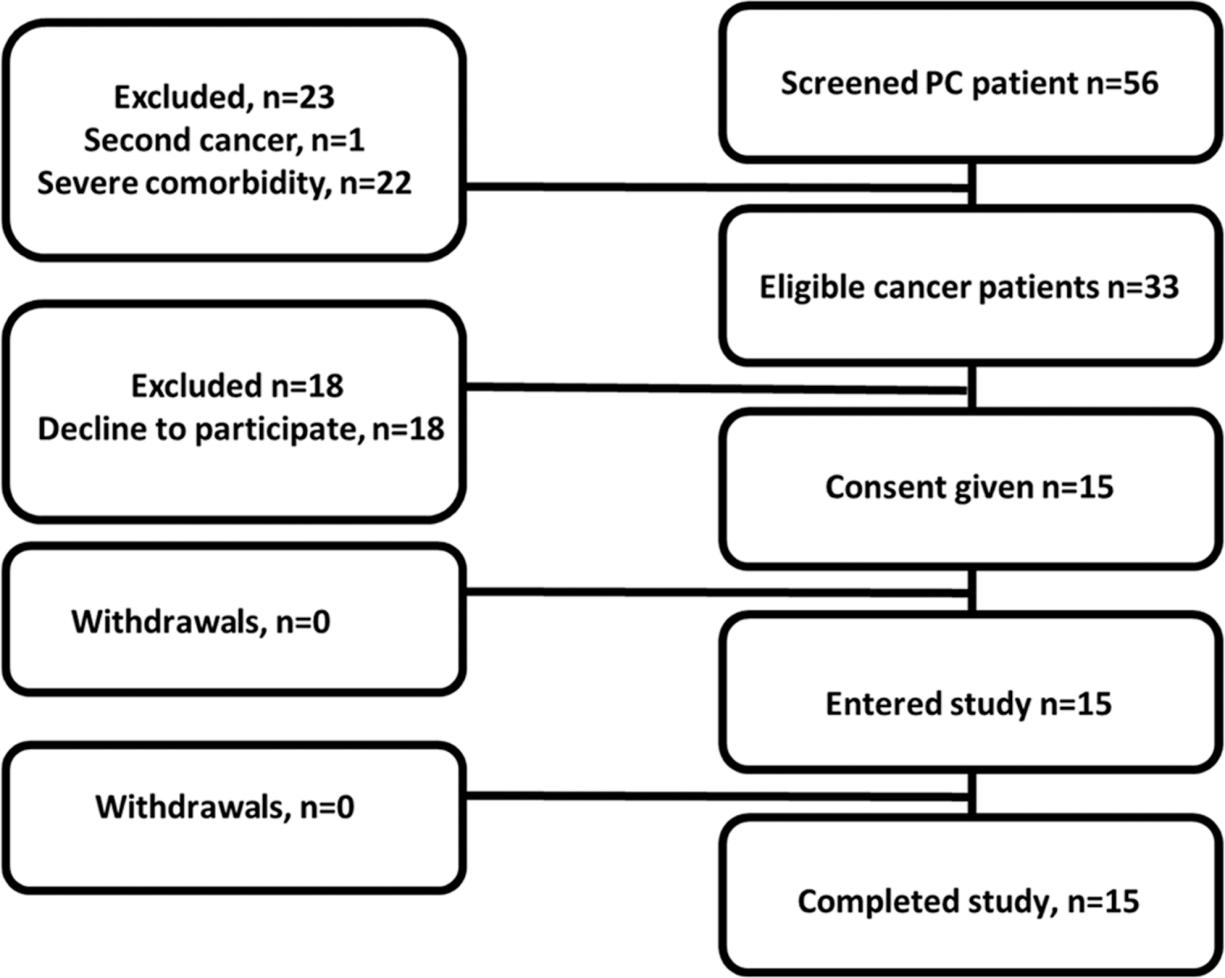
Flow diagram according to the standards for reporting diagnostic accuracy studies for PCa patients.

A single-center diagnostic phase I open-label exploratory study (ClinicalTrials.gov ID NCT05839990) was conducted at the Cancer Research Institute, Tomsk National Research Medical Center of the Russian Academy of Sciences. The initial study protocol was approved by the Institute’s Scientific Council and Board of Medical Ethics (protocol 17, approved July 21, 2023). Patients (55–75 years, 66 ± 6 years) with clinically and radiologically diagnosed and histologically verified castration-resistant or hormone-sensitive PCa were included in this study after providing written informed consent. The criteria for inclusion were the ability to undergo planned diagnostic investigations and the absence of hematological, hepatic, and renal pathologies. The exclusion criteria were a second malignancy, hepatitis B or C, HIV, or infectious diseases within the preceding 3 months, participation in other clinical studies, and a severe somatic condition caused by concomitant pathology or underlying disease. Fifteen male PCa patients were included in the study (Figure 6): 6 patients with castration-resistant (CR) and 9 patients with hormone-sensitive (HS) PCa.

An examination of the patients included measurement of PS levels, MR investigation of the pelvic organs with contrast, chest CT, and bone scans using ^99m^Tc-pyrophosphate. All patients at the time of SPECT investigation received therapy with luteinizing hormone-releasing hormone (LHRH) agonists; 12 patients additionally received therapy with second-generation antiandrogens. Six patients received bisphosphonate therapy, 4 received chemotherapy (docetaxel), and 2 (with castration-resistant PCa) received Ra-223 therapy before SPECT investigation.

### SPECT Imaging Protocol

Labeling of BQ0413 with freshly eluted technetium-99m was performed according to the protocol described in ref (27). BQ0413 (50, 100, or 150 μg) dissolved in MQ water (0.67 mg/mL) was added to a freeze-dried kit containing 5 mg of gluconic acid sodium salt (Celsus Laboratories, Geel, Belgium), 75 μg of stannous chloride (Fluka Chemika, Buchs, Switzerland), and 100 μg of EDTA (Sigma-Aldrich, Munich, Germany) freshly dissolved in PBS. Freshly eluted [^99m^Tc]Tc-pertechnetate (900–1100 MBq) was added to the mixture, and the vial was incubated at 90 °C for 60 min. The radiochemical yield was analyzed using instant thin-layer chromatography (iTLC) strips (Agilent Technologies, Santa Clara, CA) eluted with acetone (*R*_f_ = 0.1–0.2 for [^99m^Tc]Tc-BQ0413 and [^99m^Tc]Tc-TcO_2_; *R*_f_ = 0.9 for [^99m^Tc]Tc-TcO_4_^–^) and PBS (*R*_f_ = 0 for [^99m^Tc]Tc-TcO_2_; *R*_f_ = 0.9 for [^99m^Tc]Tc-BQ0413 and [^99m^Tc]Tc-TcO_4_^–^). The radioactivity distribution along the iTLC strips was measured by using an iTLC-scanner miniGITA Single (Elysia Raytest, Straubenhardt, Germany). The purity was >95%. After the analysis of [^99m^Tc]Tc-BQ0413, the labeling solution was adjusted to 10 mL using saline and sterilized by filtration.

Patients were injected with [^99m^Tc]Tc-BQ0413: 5 patients were injected with 50 μg of BQ0413 labeled with 663 ± 167 MBq, 5 patients were injected with 100 μg of peptide (653 ± 93 MBq), and 5 patients were injected with 150 μg (629 ± 89 MBq). The injections were administered as a single intravenous bolus. Imaging was performed using a hybrid system (Symbia Intevo T16) equipped with a dual-head γ camera and an integrated 16-slice CT scanner. For imaging, a low-energy high-resolution collimator was used. Patients underwent whole-body planar imaging (anterior and posterior, scan speed 12 cm/min, 1024 × 256 pixel matrix), SPECT-CT acquisition (32 projections, 30 s each, 128 × 128 pixel matrix), and low-dose CT (140 kVp, 20 mAs/slice, 512 × 512 pixel matrix) at 2, 4, 6, and 24 h after injection. The images were transferred to a Syngo.via workstation (Siemens) for analysis.

Vital signs (blood pressure, pulse, respiratory rate, temperature, and ECG) were monitored before and within 24 h of the imaging probe injection. Blood and urine samples were analyzed before and 1 day after injection. Possible side effects were evaluated within 3–7 days after injection.

### Assessment of Dosimetry

Regions of interest (ROIs) were drawn over organs and the whole body for anterior and posterior projections. The geometric means of the counts in ROIs were calculated for every time point. The activity concentrations in the blood were estimated using the ROI for the heart. The activity distributions over time were used to calculate average residence times by fitting them to a single exponential using Prism 9.2.0 (GraphPad Software, LLC, Boston, MA).^38^ Absorbed doses for individual organs, effective doses, and effective dose equivalents were calculated in an OLINDA/EXM1.1 (adult male phantom). To estimate the absorbed dose for the salivary glands, we used the sphere model in OLINDA/EXM1.1. The parotid gland volume (30 cm^3^) was taken from ref (39).The time-integrated activity was calculated using SUV_mean_ for the salivary glands.

### Statistical Analysis

Data are reported as mean ± standard deviation (*n* = 5). The median and interquartile range Me [Q1–Q3] were used to present nonparametric data. Differences of significance (one-way ANOVA, two-sided, *p* < 0.05) were tested using Prism 9.2.0.

## Data Availability

The data generated during the current study are available from the corresponding author upon reasonable request.

## Supporting Information Available

Decay-corrected activity accumulation in healthy organs over time (PDF)

## Notes

**Human Studies** A single-center diagnostic phase I open-label exploratory study (ClinicalTrials.gov ID NCT05839990) was conducted at the Cancer Research Institute, Tomsk National Research Medical Center of the Russian Academy of Sciences. The initial study protocol was approved by the Institute’s Scientific Council and Board of Medical Ethics (protocol 17, approved July 21, 2023). Patients were included in this study after providing written informed consent.

## Abbreviations

BM: bone metastases
FDA: Food and Drug Administration
FDG: [^18^F]fluorodeoxyglucose
%IA: % of injected activity
LNM: lymph node metastases
mCRPC: metastatic castration-resistant prostate cancer
MRI: magnetic resonance imaging
PCa: prostate cancer
PET: positron emission tomography
PSA: prostate-specific antigen
PSMA: prostate-specific membrane antigen
ROI: region of interest
SPECT: single-photon emission computer tomography
SUV: standardized uptake values
TRUS: transrectal ultrasound
