# Supplementary material for: Single-Photon Emission Computer Tomography Imaging of Prostate-Specific Membrane Antigen (PSMA) Expression in Prostate Cancer Patients Using a Novel Peptide-Based Probe [99mTc]Tc-BQ0413 with Picomolar Affinity to PSMA: A Phase I/II Clinical Study
Source: ACS Pharmacol Transl Sci. 2025 Feb 21;8(3):736–47. doi: 10.1021/acsptsci.4c00637 (PMC11915028; doi:10.1021/acsptsci.4c00637)
Supplement: Supplementary file 1 — pt4c00637_si_001.pdf [file pt4c00637_si_001.pdf]

Supporting Information

Single photon emission computer tomography imaging of prostate specific membrane antigen (PSMA) expression in prostate cancer patients using a novel peptide-based probe [^{99m}Tc]Tc-BQ0413 with picomolar affinity to PSMA , Phase I/II clinical study

Anna Medvedeva[1,2], Vladimir Chernov[1,2], Maria Larkina[2,3], Anstasiya Rybina[1], Roman Zelchan[1,2], Olga Bragina[1,2], Ruslan Varvashenya[2,3], Olga Zebzeeva[1], Ekaterina Bezvekhniaia[4], Vladimir Tolmachev[5], Anna Orlova[4,6*]

1 Department of Nuclear Therapy and Diagnostic, Cancer Research Institute, Tomsk National Research Medical Center, Russian Academy of Sciences, 634009 Tomsk, Russia.

2 Research Centrum for Oncotheranostics, Research School of Chemistry and Applied Biomedical Sciences, Tomsk Polytechnic University, 634009 Tomsk, Russia

3 Department of Pharmaceutical Analysis, Siberian State Medical University, 634050 Tomsk, Russia

4 Department of Medicinal Chemistry, Uppsala University, 751 23 Uppsala, Sweden

5 Department of Immunology, Genetics and Pathology, Uppsala University, 752 37 Uppsala, Sweden

6 Science for Life Laboratory, Uppsala University, 751 23 Uppsala, Sweden

* Corresponding author: Anna Orlova, anna.orlova@ilk.uu.se

‘

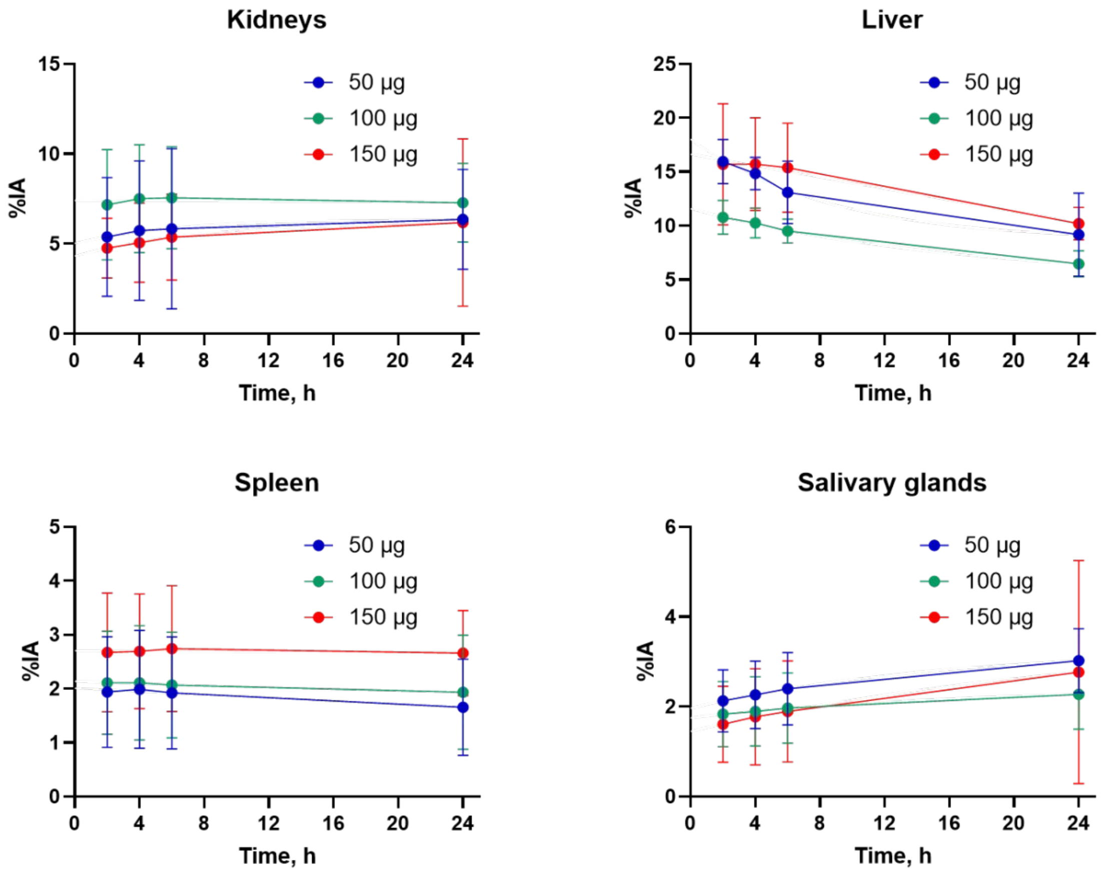

Figure S1. Decay corrected activity accumulation in healthy organs over time.
